# Effects of canagliflozin on NT-proBNP stratified by left ventricular diastolic function in patients with type 2 diabetes and chronic heart failure: a sub analysis of the CANDLE trial

**DOI:** 10.1186/s12933-021-01380-w

**Published:** 2021-09-14

**Authors:** Kenya Kusunose, Takumi Imai, Atsushi Tanaka, Kaoru Dohi, Kazuki Shiina, Takahisa Yamada, Keisuke Kida, Kazuo Eguchi, Hiroki Teragawa, Yasuchika Takeishi, Nobuyuki Ohte, Hirotsugu Yamada, Masataka Sata, Koichi Node, CANDLE Trial Investigators

**Affiliations:** 1 Department of Cardiovascular Medicine, Tokushima University Hospital, 2-50-1 Kuramoto, Tokushima, Japan; 2 Department of Medical Statistics, Osaka City University Graduate School of Medicine, Osaka, Japan; 3 Department of Cardiovascular Medicine, Saga University, Saga, Japan; 4 Department of Cardiology and Nephrology, Mie University Graduate School of Medicine, Tsu, Japan; 5 Department of Cardiology, Tokyo Medical University, Tokyo, Japan; 6 Devision of Cardiology, Osaka General Medical Center, Osaka, Japan; 7 Department of Pharmacology, St. Marianna University School of Medicine, Kawasaki, Japan; 8 Department of General Internal Medicine, Saitama Red Cross Hospital, Saitama, Japan; 9 Department of Cardiovascular Medicine, JR Hiroshima Hospital, Hiroshima, Japan; 10 Department of Cardiovascular Medicine, Fukushima Medical University, Fukushima, Japan; 11 Department of Cardiovascular Medicine, Nagoya City University East Medical Center, Nagoya, Japan; 12 Department of Community Medicine for Cardiology, Tokushima University Graduate School of Biomedical Sciences, Tokushima, Japan

**Keywords:** Canagliflozin, Type 2 diabetes mellitus, Echocardiography, Diastolic function, NT-proBNP

## Abstract

**Background:**

Identification of the effective subtypes of treatment for heart failure (HF) is an essential topic for optimizing treatment of the disorder. We hypothesized that the beneficial effect of SGLT2 inhibitors (SGLT2i) on the levels of N-terminal pro-brain natriuretic peptide (NT-proBNP) might depend on baseline diastolic function. To elucidate the effects of SGLT2i in type 2 diabetes mellitus (T2DM) and chronic HF we investigated, as a post-hoc sub-study of the CANDLE trial, the effects of canagliflozin on NT-proBNP levels from baseline to 24 weeks, with the data stratified by left ventricular (LV) diastolic function at baseline.

**Methods:**

Patients (n = 233) in the CANDLE trial were assigned randomly to either an add-on canagliflozin (n = 113) or glimepiride treatment groups (n = 120). The primary endpoint was a comparison between the two groups of the changes from baseline to 24 weeks in NT-pro BNP levels, stratified according to baseline ventricular diastolic function.

**Results:**

The change in the geometric mean of NT-proBNP level from baseline to 24 weeks was 0.98 (95% CI 0.89–1.08) in the canagliflozin group and 1.07 (95% CI 0.97–1.18) in the glimepiride group. The ratio of change with canagliflozin/glimepiride was 0.93 (95% CI 0.82–1.05). Responder analyses were used to investigate the response of an improvement in NT-proBNP levels. Although the subgroup analyses for septal annular velocity (SEP-e′) showed no marked heterogeneity in treatment effect, the subgroup with an SEP-e′ < 4.7 cm/s indicated there was an association with lower NT-proBNP levels in the canagliflozin group compared with that in the glimepiride group (ratio of change with canagliflozin/glimepiride (0.83, 95% CI 0.66–1.04).

**Conclusions:**

In the subgroup with a lower LV diastolic function, canagliflozin showed a trend of reduced NT-pro BNP levels compared to that observed with glimepiride. This study suggests that the beneficial effects of canagliflozin treatment may be different in subgroups classified by the severity of LV diastolic dysfunction.

## Introduction

Large clinical trials in patients with type 2 diabetes mellitus (T2DM) have reported that sodium-glucose co-transporter-2 inhibitors (SGLT2i) improve cardiovascular outcomes, especially the risk of hospitalization for heart failure (HF) [1–3]. In addition, a meta-analysis of these large trials in patients with T2DM also showed that SGLT2i reduced the risk of hospitalization for HF [4]. However, these trials included only a small number of patients with chronic HF and did not examine the phenotype of their HF types. In clinical guidelines, HF types are generally categorized according to symptoms and systolic function stratified by ejection fraction (EF) [5]. In patients with HF and a reduced EF (HFrEF), neurohormonal antagonists can reduce the risk of cardiovascular events. However, these established treatments for HFrEF have shown no efficacy in trials of HF with preserved EF (HFpEF) [6, 7]. Recently, several researchers reported that advanced imaging can identify subtypes in which treatment was effective [8, 9]. An advancing area in this research field is the application of artificial intelligence to carry out detailed phenotyping using multi-omics data, which enables stratification of early-stage diseases and assessment of prognosis [10, 11]. Identification of effective subtypes of HF treatment is therefore an essential research topic for optimizing treatment of HF [12].

We undertook a randomized trial that compared treatment between the SGLT2i, canagliflozin and glimepiride, with the primary objective of assessing the effect of canagliflozin on N-terminal pro-brain natriuretic peptide (NT-proBNP) levels in T2DM patients with chronic HF [13]. This study did not reach the predefined primary endpoint after 24 weeks of treatment with canagliflozin vs. glimepiride (non-inferiority for the group ratio of percentage change in NT-proBNP level). Interestingly, the reduction in NT-proBNP levels caused by canagliflozin treatment was more obvious with HFpEF than with HFrEF. Therefore, we hypothesized that the effect on NT-proBNP might depend on baseline cardiac function. To elucidate the effects of SGLT2i in T2DM, we investigated the effects of canagliflozin on NT-proBNP levels stratified by baseline left ventricular diastolic function as a sub-study of the CANDLE trial.

## Methods

### Study design and population

**Fig. 1:**
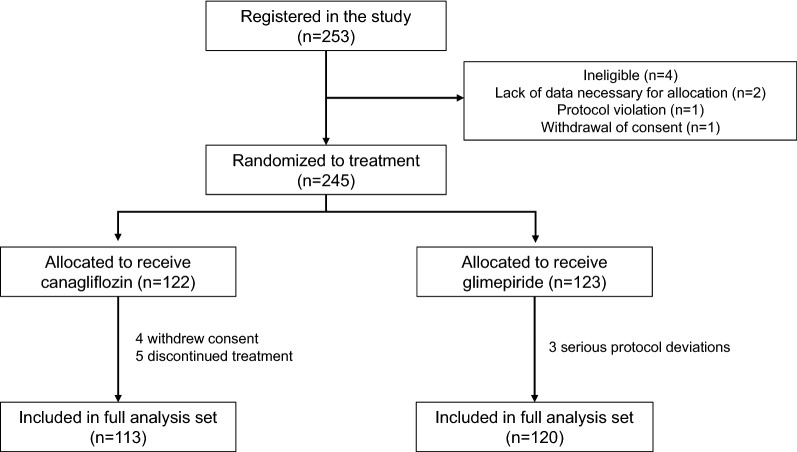
Flow diagram of the patient selection process

This was a post hoc analysis of the CANDLE trial (UMIN000017669). The details of the CANDLE study design have been published elsewhere [14]. Briefly, the study was a multicenter, randomized, prospective, open-label, blinded-endpoint trial in 34 institutions in Japan. The ethical committees of each participating institution approved the study protocol, with written, informed consent for participation in the study being obtained from all subjects. After exclusion of ineligible patients, a total of 245 patients with T2DM and chronic HF were randomized in the study between August 2015 and June 2017. Patients were assigned randomly using a 1:1 ratio to either add-on canagliflozin treatment (canagliflozin group, n = 122) or glimepiride treatment (glimepiride group, n = 123). After exclusion of patients who did not provide consent to participate in the study, discontinued treatment, or had serious protocol deviations, 113 and 120 patients administered canagliflozin or glimepiride, respectively were included in the analysis dataset (Fig. 1). The primary endpoint of the CANDLE study was the change in NT-proBNP levels at 24 weeks after treatment randomization. Echocardiography was performed at baseline (canagliflozin group, n = 105, and glimepiride group, n = 112) with the results used to stratify the patients into subgroups for data analysis.

### Echocardiographic assessment

Echocardiography was performed in a standard manner using a commercially available ultrasound diagnostic machine, with the various hemodynamic parameters measured at each institution. The recordings and measurements were carried out according to the guidelines issued by American Society of Echocardiography [15]. LV ejection fraction (LVEF) was measured and calculated from the apical two- and four-chamber views using bi-plane disk methods. Transmitral flow (TMF) velocity was recorded from the apical long-axis or four-chamber view. The peak early diastolic (E) velocities were measured. The mitral annular motion velocity pattern was recorded from the apical four-chamber view with the sample volume placed at the septal side of the mitral annulus measured using pulsed tissue Doppler echocardiography. Early diastolic (e′) peak velocities were measured and the ratio of E to e′ (E/e′) then calculated. These velocity parameters were used as markers of LV diastolic function.

### Laboratory examination

Blood samples were collected at baseline and after 12 and 24 months. The serum levels of NT-proBNP were measured in a centralized laboratory (SRL Co. Tokyo, Japan) using an electrochemiluminescence immunoassay (ECLIA) and nephelometry.

### Statistical analysis

The baseline characteristics were expressed using frequencies with percentages for categorical variables and means with standard deviations for continuous variables. Chance imbalances in baseline characteristics between the two groups were assessed using standardized difference (std diff), with a value < 0.2 indicating adequate balance in the groups. All statistical analyses were performed according to the intention-to-treat principle. For evaluation of the follow-up HbA1c levels, the analyses were performed using linear mixed models with adjustment for the baseline value. The mean values with 95% confidence intervals (CI) at 4, 12, and 24 weeks for both groups were plotted and then compared. The NT-proBNP analyses were performed using linear models on the logarithmic scale, with adjustment for the baseline value. Changes in the geometric means and 95% CI from baseline to 24 weeks for both treatment groups and the ratio of the geometric means at 24 weeks between treatment groups were calculated. To examine the consistency of the treatment effect across the values of SEP-e′ and E/SEP-e′, two types of analyses were performed. In the first analysis, subgroup analysis was performed for the three groups defined by tertiles of SEP-e′ or E/SEP-e′ values at baseline for all patients. In the second analysis, SEP-e′ or E/SEP-e′ values were included in the model of NT-proBNP at 24 weeks using the restricted cubic spline function. The estimated changes in the geometric means for both treatment groups were plotted against SEP-e′ or E/SEP-e′ values. All analyses were performed using R software, version 3.6.3 (R Foundation for Statistical Computing). Statistical significance was defined as a *p* value < 0.05.

## Results

### Baseline clinical characteristics

The baseline clinical characteristics of the canagliflozin and control groups are shown in Table 1. Approximately 40% of the patients in the two groups had hypertension, with blood pressure being well controlled in both groups. There was no significant difference in the standardized difference in any of the variables examined between the two groups at baseline, with the exception of heart rate which was marginally higher in the canagliflozin group than in the glimepiride group (standardized difference = 0.226). The 1/3 and 2/3 percentiles in all the patients at baseline were 4.7 cm/s and 6.4 cm/s for SEP-e′ and 10.2 and 13.9 for E/SEP-e′, respectively. The number of patients in each subgroup defined by these values are described in Table 2. Patients with missing data for SEP-e′ or E/SEP-e′ were excluded from the subgroup analyses.

**Table 1:** Clinical characteristics of the patients in the two treatment groups

	Canagliflozin	Glimepiride	Std diff
Number	113	120	
Clinical background			
Age, yr	69 ± 9	69 ± 9	0.058
Male	88 (78)	86 (72)	0.143
Body mass index kg/m^2^	25 ± 3	26 ± 4	0.094
Systolic BP, mmHg	125 ± 14	125 ± 18	0.021
Heart rate, bpm	75 ± 12	72 ± 13	0.226
Clinical history			
Hypertension	49 (43)	53 (44)	0.016
Dyslipidemia	46 (41)	54 (45)	0.087
Myocardial infarction	32 (28)	24 (20)	0.195
Angina pectoris	24 (21)	27 (23)	0.031
Coronary artery bypass grafting	12 (11)	11 (9)	0.049
NYHA			
I	72 (64)	76 (63)	0.008
II	39 (35)	40 (33)	0.025
III	2 (2)	3 (3)	0.051
Unknown	0 (0)	1 (1)	0.130
Medications for non-diabetic conditions			
ACEi/ARB	89 (79)	88 (73)	0.127
Beta blocker	82 (73)	82 (68)	0.093
MRA	42 (37)	44 (37)	0.010
Diuretic	46 (41)	53 (44)	0.070
Statin	87 (77)	86 (72)	0.122
Anti-platelet or anti-coagulant	71 (63)	66 (55)	0.160
Medication for diabetes			
Insulin	4 (4)	3 (3)	0.061
Metformin	18 (16)	26 (22)	0.147
Alpha-glucosidase inhibitor	16 (14)	24 (20)	0.156
DPP-4 inhibitor	64 (57)	63 (53)	0.083
GLP-1RA	1 (1)	1 (1)	0.006
Echocardiography			
TMF-E	73.6 ± 25.7	71.9 ± 30.58	0.058
SEP-e′	6.27 ± 2.87	5.75 ± 2.28	0.199
E/e′	11.38 ± 5.15	11.59 ± 5.29	0.040
EF	56.67 ± 14.47	56.64 ± 14.43	0.002

The data are expressed as number of patients (percentage), mean ± SD, or median (interquartile range). A standardized difference (std diff) of < 0.2 indicates adequate balance

Abbreviations: BP, blood pressure; NYHA, New York Heart Association; ACEi/ARB, angiotensin-converting-enzyme inhibitor/angiotensin II receptor blocker; MRA, mineralocorticoid receptor antagonist; DPP-4, dipeptidyl peptidase 4; GLP-1RA, glucagon like peptide-1 receptor agonist. TMF-E, early diastolic transmitral flow; SEP-e′, septal early diastolic mitral annular velocity; EF, ejection fraction

**Table 2:** Number of patients in each subgroup defined by SEP-e′ and E/SEP-e′

Subgroups	Number of patients	
	Canagliflozin	Glimepiride
SEP-e′: < 4.7	33	40
SEP-e′: 4.7–6.4	36	36
SEP-e′: ≥ 6.4	36	36
SEP-e′: missing	8	8
E/SEP-e′: < 10.2	34	38
E/SEP-e′: 10.2–13.9	35	37
E/SEP-e′: ≥ 13.9	34	37
E/SEP-e′: missing	10	8

See abbreviations in Table 1

**Fig. 2:**
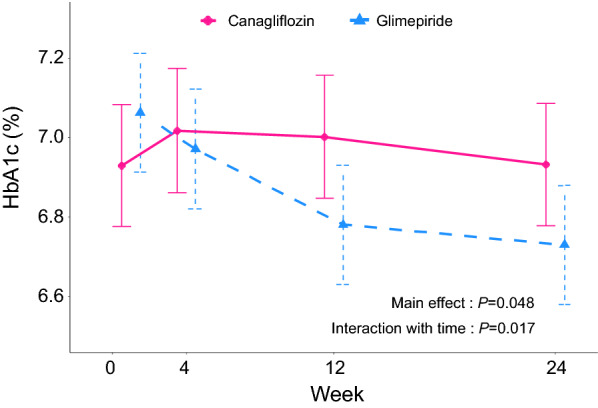
Changes in HbA1c at 4, 12, and 24 weeks in the two treatment groups

Figure 2 showed the changes in HbA1c during the study period. A larger reduction in HbA1c level was observed in the glimepiride group (*P* for main effect = 0.048 and *P* for interaction with time = 0.017). The final HbA1c levels were 6.93 mg/dL and 6.73 mg/dL in the canagliflozin and glimepiride groups, respectively.

### Effect on NT-proBNP level

**Fig. 3:**
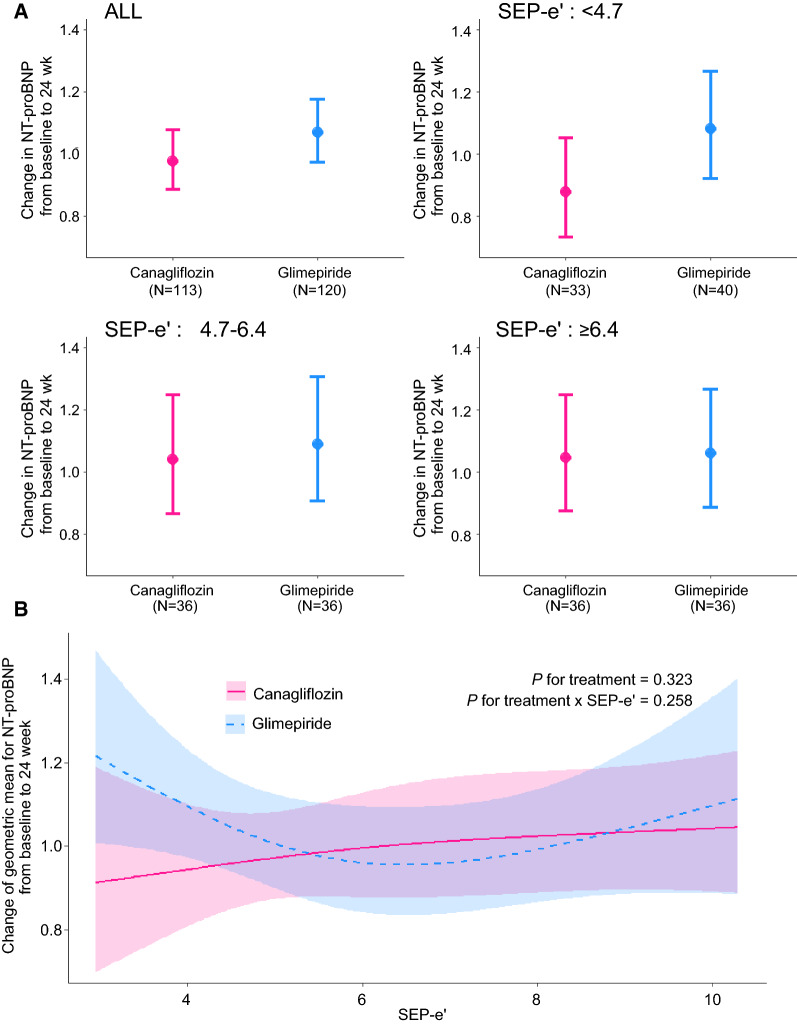
Changes in the geometric means of NT-proBNP from baseline to 24 weeks stratified by septal e′ (SEP-e′) in the two treatment groups. **A** Three groups defined by tertiles of SEP-e′. **B** A linear model using raw SEP-e′ values with restricted cubic spline function

At baseline, the geometric mean of NT-proBNP level was 217.4 (95% CI 169.3–279.1) in the canagliflozin group and 211.8 (95% CI 166.4–269.6) in the glimepiride group. At 24 weeks, the geometric mean of NT-proBNP level was 212.8 (95% CI 165.5–273.5) in the canagliflozin group and 225.8 (95% CI 177.2–287.8) in the glimepiride group. The change from baseline to 24 weeks was 0.98 (95% CI 0.89–1.08) in the canagliflozin group and 1.07 (95% CI 0.97–1.18) in the glimepiride group (Fig. 3A). The ratio of change with canagliflozin/glimepiride was 0.93 (95% CI 0.82–1.05).

**Fig. 4:**
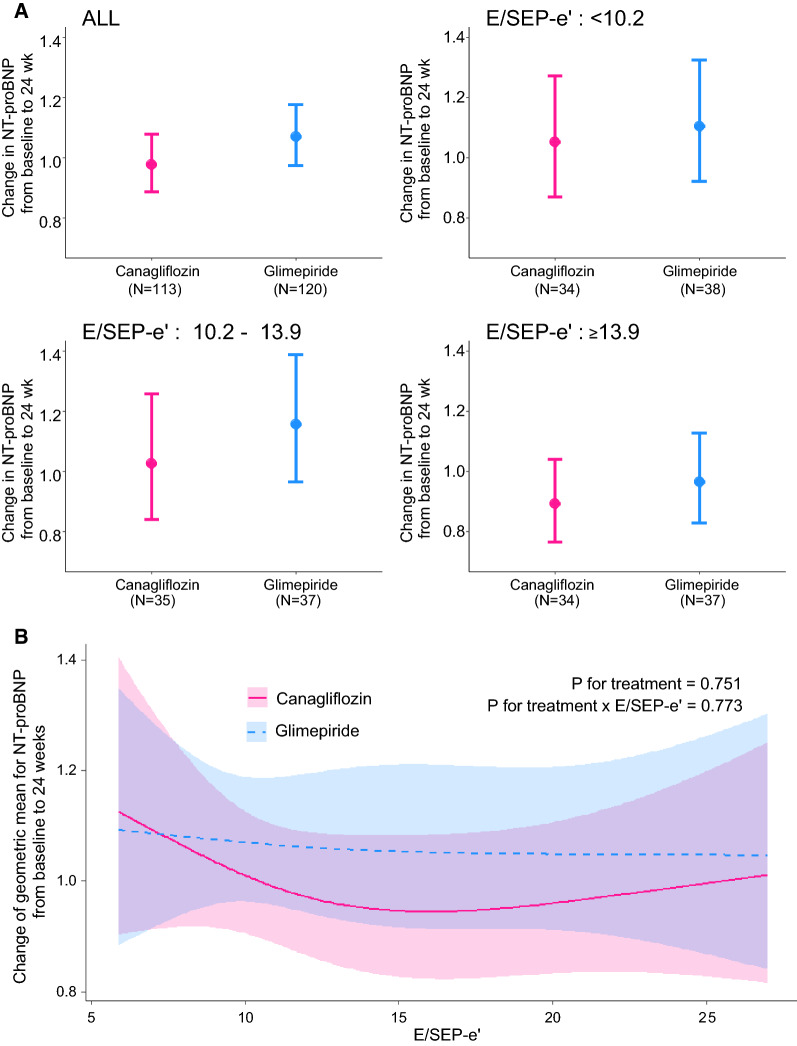
Changes in the geometric means of NT-proBNP from baseline to 24 weeks stratified by E/e′ in the two treatment groups. **A** Three groups defined by tertiles of E/SEP-e′. **B** A linear model using raw E/SEP-e′ value with restricted cubic spline function

The results of the subgroup analyses are summarized in Table 3 and plotted in Figs. 3A and 4A. No marked heterogeneity in the treatment effect was observed.

**Table 3:** Change in geometric means of NT-proBNP in each subgroup

Subgroups	Change in GMs of NT-proBNP at 24 wk (95% CI)		Ratio of change in GM (95% CI)	*P* value
	Canagliflozin group	Glimepiride group		
All	0.98 (0.89 to 1.08)	1.07 (0.97 to 1.18)	0.93 (0.82 to 1.05)	0.244
SEP-e′: < 4.7	0.88 (0.73 to 1.05)	1.08 (0.92 to 1.27)	0.83 (0.66 to 1.03)	0.083
SEP-e′: 4.7 to 6.4	1.04 (0.87 to 1.25)	1.09 (0.91 to 1.31)	1.02 (0.81 to 1.28)	0.893
SEP-e′: ≥ 6.4	1.04 (0.87 to 1.25)	1.06 (0.89 to 1.27)	1.00 (0.78 to 1.28)	0.992
E/SEP-e′: < 10.2	1.05 (0.87 to 1.27)	1.01 (0.92 to 1.33)	0.95 (0.74 to 1.22)	0.685
E/SEP-e′: 10.2 to 13.9	1.03 (0.84 to 1.26)	1.16 (0.97 to 1.39)	0.98 (0.75 to 1.26)	0.849
E/SEP-e′: ≥ 13.9	0.89 (0.76 to 1.04)	0.96 (0.83 to 1.13)	0.92 (0.76 to 1.12)	0.413

GM: geometric means

See abbreviations in Table 1

Among the subgroups defined by tertiles of SEP-e′ values, the first tertile of SEP-e′ (< 4.7 cm/s) appeared to be associated with lower NT-proBNP levels in the canagliflozin group compared with those observed in the glimepiride group (ratio of change with canagliflozin/glimepiride: 0.83, 95% CI 0.66–1.03). Although this difference was not statistically significant, the same trend was suggested in the linear model using raw SEP-e′ values (Fig. 3B).

Among the subgroups defined by tertiles of E/SEP-e′ values, the third tertile of E/SEP-e′ (≥ 13.9) also appeared to be associated with lower NT-proBNP levels in the canagliflozin group compared with those observed in the glimepiride group (ratio of change with canagliflozin/glimepiride: 0.95, 95% CI 0.74–1.22). Although this difference was not statistically significant, the same trend was observed in the linear model using raw E/SEP-e′ values (Fig. 4B).

## Discussion

This study was a sub-study of the CANDLE trial and assessed the effects of canagliflozin on changes in NT-proBNP levels stratified by LV diastolic function from baseline to 24 weeks. This prospective, randomized study provides new insights into the understanding of SGLT2i on NT-proBNP. We observed that canagliflozin treatment tended to reduce NT-proBNP levels to a greater extent than glimepiride in patients with diastolic dysfunction (decreased e′ and elevated E/e′). The results of this study may help to identify subtypes of HF in which SGLT2i is more effective and therefore worthy of further investigation.

### Mechanisms of SGLT2i on NT-proBNP

Many investigators have shown that SGLT2i provides beneficial cardiovascular effects as a consequence of changes in several pathways [16, 17]. There are several potential mechanisms for this phenomenon: (1) a diuretic effect of SGLT2i, (2) hyperketonemia that switches myocardial fuel usage from glucose to ketone bodies and free fatty acids, resulting in more efficient ATP production [18], and (3) SGLT2i inhibiting cardiac Na+-H+ exchanger (NHE), thereby reducing intracellular calcium and increasing mitochondrial Ca2+, which restores mitochondrial function and redox state and activates ATP production [19]. Support for these potential mechanisms was obtained by the findings of studies in animal models such as the use of SGLT2i to attenuate myocardial oxidative stress and fibrosis in diabetic mice heart, or improve coronary microvascular function and cardiac contractility in a pre-diabetic mice model [20, 21]. Based on these beneficial cardiovascular effects, the use of SGLT2i appears to lower NT-proBNP levels. A small number of randomized clinical trials have investigated the effect of SGLT2i on NT-proBNP levels as a measure of the treatment impact on HF [22]. More recently, the use of dapagliflozin over 12 weeks did not affect NT-proBNP levels in patients with HFrEF, although it increased the proportion of patients who experienced clinically meaningful improvements in HF-related health status or natriuretic peptide levels [23]. Therefore, the effect of SGLT-2i on NT-proBNP remains controversial and more valid subtypes need to be identified.

### Impact of SGLT2i in HF subtypes

It has long been recognized that cardiovascular medication is well established in patients with HFrEF but not in those with HFpEF. The identification of subtypes in which these drugs are useful is therefore an important clinical issue in the field of HF. Several analyses showed that pre HFrEF status and HF mid-range EF patients had a better prognosis, similar to that of patients with HFrEF when therapies recommended in guidelines were used [24, 25]. From this perspective, the administration of cardioprotective medications in patients with subclinical myocardial dysfunction may have greater beneficial effects on cardiovascular function compared to that in patients with normal cardiac function. In addition, despite the absence of overt structural heart disease or symptoms of HF, patients with diabetes are considered as “stage A HF” because they have an increased risk of developing HF [26]. These patients often demonstrate subclinical myocardial dysfunction, which can be diagnosed by the presence of diastolic dysfunction and LV hypertrophy [27]. Subclinical LV dysfunction may be a key marker for the effects of SGLT2i. In our study, canagliflozin treatment tended to reduce NT-proBNP levels to a greater extent than glimepiride in patients with LV diastolic dysfunction. These findings suggest that patients with impaired LV diastolic function may also benefit from administration of SGLT2i. This finding may provide profound insights into the effects of SGLT2i according to HF phenotypes.

The effect on NT-proBNP has been examined in previous studies, however the results are controversial. One possible explanation is that the efficacy of SGLT2i also depends on the cardiac function at baseline because the efficacy of cardioprotective drugs including β-blocker and renin–angiotensin–aldosterone system inhibitor depends on EF and the HF guidelines recommended EF guided treatment strategies. Although a recent meta-analysis demonstrated that treatment with a SGLT2i was associated with significant improvements in plasma NT-proBNP concentrations in patients with T2DM, irrespective of the presence of HF, the LVEF level was improved only in HFrEF [28]. In this context, we need an additional assessment of echocardiographic parameters to predict the cardiovascular favor effects of SGLT2i. Our study is the first to suggest that baseline diastolic function may be an important role in the prediction of SGLTi efficacy on plasma NT-proBNP.

Dapagliflozin was found to be associated with improvement of left ventricle (LV) longitudinal myocardial function leading to an improvement of LV diastolic function of T2DM patients with stable heart failure (HF) [29]. Its beneficial effect possibly targets coronary endothelium [30]. Furthermore, empagliflozin ameliorates diastolic function in a nondiabetic HF porcine model, mitigating histological and molecular remodeling, and reducing both LV and cardiomyocyte stiffness [31]. Therefore, other SGLT2 inhibitors may present similar effects as those described in the present paper.

### Clinical implication

Although evidence of the beneficial effects of SGLT2i on HF is accumulating, the characterization of cardiac function that would identify the patient groups that would benefit from administration of SGLT2i has not been examined in detail. Diastolic dysfunction may be a good indicator for identifying the beneficial effects on NT-proBNP levels in T2DM. Unfortunately, we were unable to show a strong correlation between diastolic dysfunction and an improvement in NT-proBNP levels in the present study, although it is possible this relationship may be shown in further trials.

### Limitation

This study was a sub-analysis of the CANDLE study and echocardiography was not performed in all subjects. Therefore, the number of study subjects was relatively small and included only Japanese patients. The study was a post hoc analysis of the CANDLE trial that was not designed specifically or powered to perform subgroup analysis according to baseline echocardiographic data. Although this method for analyzing NT-proBNP concentrations has been used in several other clinical trials on HF that assessed the interventional impact on NT-proBNP concentration as a surrogate endpoint of HF treatment [32, 33], it is still controversial whether measuring natriuretic peptides is sufficient to identify a clinically meaningful treatment effect [34, 35]. Multiple parameters of HF-related health status should be treated in future studies. In the recent American Society of Echocardiography/European Association of Cardiovascular Imaging (ASE/EACVI) recommendations [15], diastolic dysfunction was assessed using LA volume index and peak tricuspid regurgitation velocity. However, this trial has been designed to gather the limited echocardiographic variables (TMF-E, SEP-e′, E/e′, and EF), thus, we used only these variables in this study. Although the participant’s background treatment will be, in principle and if possible, unchanged during the trial interval, the other medications might affect on NT-proBNP in this study. Unfraternally, we did not gather the detail of medication modifications during the study period.

## Conclusion

To our knowledge, this is the first prospective cohort study suggesting that the beneficial effects of canagliflozin treatment can differ in subtypes of HF stratified according to LV diastolic dysfunction.

## Data Availability

The datasets analyzed during the current study are available from the corresponding authors on reasonable request (contact via candle-sub@clin-med.org).

## Abbreviations

T2DM: Type 2 diabetes
SGLT2i: Sodium-glucose co-transporter-2 inhibitors
HFrEF: Heart failure with reduced ejection fraction
HFpEF: Heart failure with preserved ejection fraction
NT-proBNP: N-terminal pro-brain natriuretic peptide
